# Anti-HMGB1 antibody reduces weight gain in mice fed a high-fat diet

**DOI:** 10.1038/nutd.2015.11

**Published:** 2015-06-15

**Authors:** V N Montes, S Subramanian, L Goodspeed, S A Wang, M Omer, A Bobik, K Teshigawara, M Nishibori, A Chait

**Affiliations:** 1 Division of Metabolism, Endocrinology and Nutrition, Department of Medicine, University of Washington, Seattle, WA, USA.; 2 BakerIDI Heart and Diabetes Institute, Melbourne, Australia; 3 Department of Pharmacology, Okayama University Graduate School of Medicine, Dentistry, and Pharmaceutical Sciences, Okayama, Japan

## Abstract

Insulin resistance in obesity is believed to be propagated by adipose tissue and liver inflammation. HMGB1 is a multifunctional protein that is pro-inflammatory when released from cells. It has been previously demonstrated that anti-HMGB1 antibody reduces atherosclerotic lesion pro-inflammatory cells and progression of atherosclerosis in a mouse model. To test the potential beneficial role of blocking HMGB1 in adipose tissue and liver inflammation in mice fed an obesogenic diet, we administered anti-HMGB1 antibody to C57Bl/6 mice fed a high (60%)-fat diet. The mice were treated with weekly injections of an anti-HMGB1 antibody or anti-KLH antibody (isotype control) for 16 weeks. Mice that received the anti-HMGB1 antibody gained less weight than the control-treated animals. Anti-HMGB1 treatment also reduced hepatic expression of TNF-alpha and MCP-1, molecules that promote inflammation. However, adipose tissue inflammation, as measured by gene expression analyses and immunohistochemistry, did not differ between the two groups. There also were no differences in glucose or insulin tolerance between the two groups. When feeding mice a high-fat diet, these data suggest that HMGB1 may have a crucial role in weight gain and liver inflammation.

## Introduction

Obesity is characterized by elevated body mass index and is frequently accompanied by systemic and tissue-specific inflammation.^1^ There is not just a change in adipose tissue mass, but also a change in the inflammatory nature of the tissue, characterized by an infiltration of pro-inflammatory cells such as dendritic cells, macrophages, CD4+ and CD8+ T cells, among others.^2^ Current dogma suggests that adipose tissue inflammation leads to insulin resistance, which increases cardiovascular disease risk.^3^ In addition, obesity-associated fatty liver leads to increased hepatic pro-inflammatory cytokine production.^4^ Atherosclerotic lesions demonstrate a similar profile of pro-inflammatory cellular components as seen in adipose tissue in obesity.^5^ Hence, it is possible that treatments that affect inflammation in the vascular wall may have similar effects on comparable types of adipose tissue inflammation.

HMGB1 is a molecule that has a dual function; it is an integral part of chromatin structure acting to facilitate transcription, but when released from a cell can trigger potent pro-inflammatory reactions.^6^ Intracellularly, it binds to DNA to allow interaction with transcription factors. However, it can be passively released from cells through cell death and actively secreted by pro-inflammatory cells such as macrophages, and is thought to interact with multiple pro-inflammatory receptors including the receptor for advanced glycation end products and TLR2/4.^7^ It is this secretory role that has led to an interest in the ability to harness inflammation through neutralization of circulating HMGB1.^8^ HMGB1 has been detected in atherosclerotic lesions in apoE-deficient mice.^9^ Treatment with an anti-HMGB1 antibody led to a reduction in many inflammatory cell types such as dendritic cells, macrophage and CD4+ T cells in atherosclerotic lesions, as well as decreased lesion size in apoE-deficient mice.^10^ As HMGB1 appears to have a role in tissue inflammation, we hypothesized that neutralization of HMGB1 with an anti-HMGB1 antibody would reduce adipose tissue inflammation and insulin resistance in mice fed a high-fat diet (HFD).

## Methods

### Reagents

Anti-HMGB1 and anti-KLH (inactive IgG2a isotype control) antibodies were obtained from Dr. Masahiro Nishibori, Okayama University Graduate School of Medicine, Japan.

### Animals and diet

Eight-week-old male C57BL/6 mice were purchased from Jackson Laboratories (Sacramento, CA, USA). Two groups of mice (*n*=8 per group) were fed a HFD that provides 60% calories from fat (D12492, Research Diets, Newbrunswick, NJ, USA) for 16 weeks and either received intraperitoneal injection of anti-HMGB1 or anti-KLH antibody (400 μg per injection) twice per week for the duration of the diet, which was the dose used in the apoE-deficient mouse study.^10^ Body composition and energy expenditure were assessed non-invasively in unanesthetized mice using the University of Washington's Nutrition Obesity Research Center's (NORC) Energy Balance and Glucose Metabolism Core Laboratory, as previously described.^11^

### Blood chemistry

Blood (50 μl) was collected from the retro-orbital sinus after a 4 h fast before commencement of the diet and then every 4 weeks until sacrifice. Plasma glucose, triglycerides, cholesterol and serum amyloid A were measured as described previously.^12, 13^

### Adipose tissue—stromal vascular fraction separation

Epididymal white adipose tissue (EWAT) was excised at the time of sacrifice and the stromal vascular cell (SVC) fraction separated as previously described.^14^ The SVC fraction and whole EWAT were used for gene expression analysis.

### Histological and immunohistochemical characterization of tissues and adipocyte sizing

Tissues were preserved in 10% formalin for 24 h and fixed in paraffin. Sections were utilized for hematoxylin and eosin and Movat's pentachrome staining as described previously.^15^ Adipose tissues and liver were also stained with the macrophage-specific antibody Mac2. Immunohistochemical analysis was performed as described previously.^16^ Adipocyte number and cross-sectional area were determined using computer image analysis with Image J software (U.S. National Institutes of Health, Bethesda, MD, USA) with size criteria of 250–25 000 μm^2^ and circularity of 0.3–1.0. These analyses were performed by the University of Washington's NORC Adipose Tissue and Lipid Biology Core Laboratory.

### Real-time quantitative PCR of adipose tissue and liver

Total RNA from harvested tissue (adipose tissue, SVCs and liver) was extracted with a QIAGEN (Valenica, CA, USA) kit as described,^14^ with co-amplification of messenger RNA for mouse *Gapdh* as the housekeeping gene. Relative expression was assessed by the comparative C_T_ method.

### Glucose and insulin tolerance testing

Glucose tolerance testing was performed after a 4 h fast by sampling at 0, 30, 60 and 120 min by tail nicking after intraperitoneal glucose (1 mg kg^−1^) injections a 13 weeks on diet. Insulin tolerance testing was performed at 14 weeks on diet as previously described.^17^

### Statistics

Data are expressed as means±s.e.m. Mean values were compared using Student's *t-*test where *P*<0.05 was considered statistically significant.

## Results

### Weight gain is reduced in mice receiving anti-HMGB1 antibody

**Figure 1:**
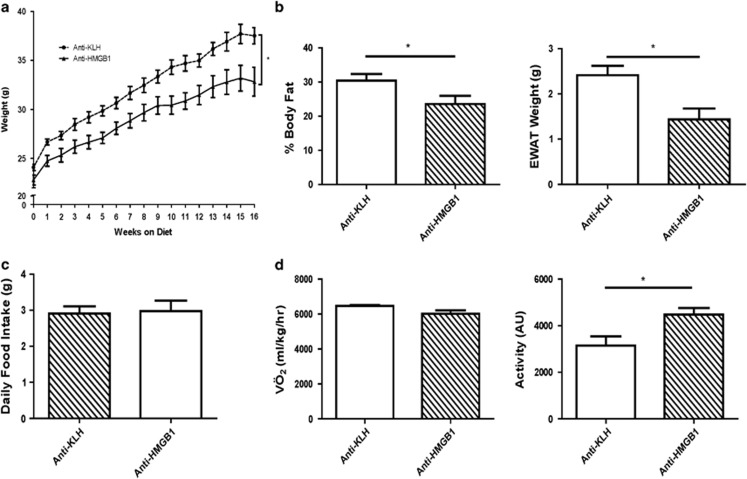
Body and tissue weights, food intake and energy expenditure. (**a**) Body weights differed significantly over the 16-week study, starting as early as 1 week (*n*=8). (**b**) Body fat percentage and epidydimal adipose tissue weights were lower towards the end of the study in mice receiving the HMGB1 antibody (*n*=8). (**c**) Food intake was the same in both the groups (*n*=4). (**d**) Energy expenditure as measured by VÖ_2_ was not different between the groups, but activity during the light cycle was increased in mice given anti-HMGB1 antibody (*n*=4; *P<0.05).

The 60% HFD resulted in significant weight gain over 16 weeks. However, mice receiving the anti-HMGB1 antibody gained significantly less weight over the study duration (*P*<0.05; Figure 1a). Body composition analysis revealed a significant difference in the body fat composition in the anti-HMGB1 antibody-treated mice compared with control mice with a corresponding difference in EWAT weights (*P*<0.05; Figure 1b). Average adipocyte size was significantly smaller in anti-HMGB than control mice (3531 vs 4957 μm^2^; *P*<.0001). Liver weights did not differ (data not shown) and food intake was similar in both the groups of mice (Figure 1c). Energy expenditure as measured by VÖ_2_ did not differ during the dark or light cycles, but activity during the light cycle was increased in the mice treated with anti-HMGB1 (*P*<0.05; Figure 1d). No change was observed in plasma cholesterol or triglycerides (data not shown). Glucose and insulin tolerance curves did not differ (data not shown).

### Pro-inflammatory cytokine expression was reduced in the liver but not adipose tissue of mice treated with anti-HMGB1 antibody

**Figure 2:**
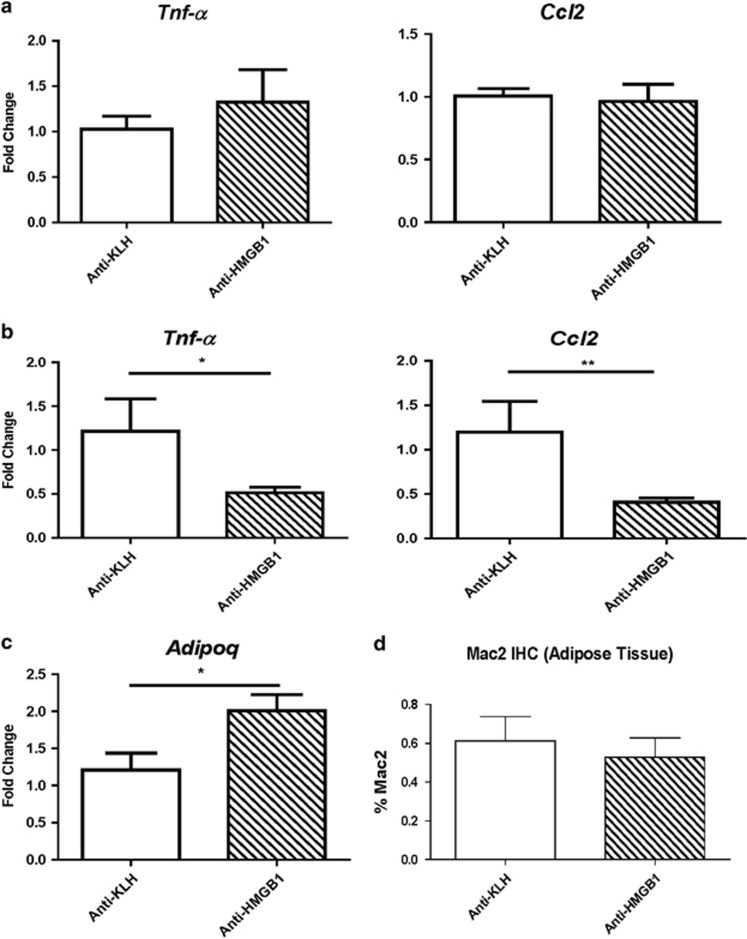
Gene expression of liver and adipose tissue, and immunohistochemistry of adipose tissue. (**a**) Liver gene expression showed decreased expression of *Tnf-α* and *Ccl2* (*n*=8). (**b**) Adipose tissue showed no changes in the expression of these two genes, (**c**) but adiponectin expression was reduced in mice treated with the HMGB1 antibody (*n*=8). (**d**) Immunohistochemistry staining for macrophages in adipose tissue did not differ between the two groups (*n*=4; *P<0.05, **P<0.01).

*Tnf-α* and *Ccl2* gene expression were reduced in the liver (Figure 2a), suggesting a decrease in inflammation. Gene expression in the adipose stromal vascular fraction did not show significant changes in pro-inflammatory cytokines such as *Tnf-α* and *Ccl2* (Figure 2b). Despite these findings, the gene expression of adiponectin was increased in whole EWAT of the mice treated with anti-HMGB1 antibody (Figure 2c). Whole EWAT did not show differences in the expression of genes associated with beta-oxidation such as *Ucp1*, *Cpt1α* or *Ppar-α*; nor were there differences in *Ppar-γ* or *Ppargc1α* (data not shown). There was no difference in staining for Mac2, a pan macrophage marker in EWAT (Figure 2d). Serum amyloid A, an inflammatory marker, was not significantly different (data not shown).

## Discussion

In this study, we have shown that weight gain in mice on a HFD is reduced by the administration of an anti-HMGB1 antibody. The antibody also differentially affected expression of liver pro-inflammatory molecules. The treatment caused a trend towards altered body fat composition with a corresponding significant decrease in the mass of epididymal fat pads. The mechanism of reduced weight gain is incompletely understood in this setting. We do show an increase in activity during the light cycle of an energy expenditure study but no increase in overall energy expenditure. The mice ate the same amount of food, which suggests that the mechanism is unrelated to food intake. There were no differences in genes associated with fatty acid utilization in adipose tissue. The study was limited by the total number of mice evaluated, particularly for the energy expenditure studies.

Reduced weight gain or weight loss is usually accompanied by improved glucose and insulin tolerance, but we surprisingly did not observe this. Another factor that is associated with insulin resistance is adipose tissue inflammation.^18^ Although anti-HMGB1 treatment reduced liver inflammation, indicated by reduced expression of *Tnf-α* and *Mcp-1*, we were unable to show an anti-HMGB1 antibody-induced improvement in adipose tissue inflammation induced by a HFD. This lack of effect on adipose tissue inflammation contrasted with the study in which administration of an anti-HMGB1antibody of led to a reduction in macrophage content of atherosclerotic lesions in apoE-deficient mice.^10^ This raises the question of whether HMGB1 has a different role in adipose versus liver and vascular inflammation. The current dogma suggests that the lack of adipose inflammation improvement may be one reason that we did not see the requisite changes in glucose metabolism variables. With an improvement in liver inflammation based on limited gene expression analysis, we would have expected this to be associated with improved glucose metabolism, but that did not occur. It may be that adipose inflammation may override any beneficial effects from reduced weight gain and liver inflammation. Also, there may be other unknown aspects of HMGB1 neutralization that may account for this as well. Interestingly, we did show an increase in adiponectin gene expression in whole adipose tissue, but this may not reflect post-translational adiponectin protein expression, secretion and/or degradation.

Overall, neutralization of HMGB1 appears to reduce weight gain and liver inflammation in mice fed an obesogenic diet. To assess the strategy of HMGB1 neutralization as an obesity treatment, further studies to expand the results found here are warranted.

## References

[bib1] Hotamisligil GS Inflammation and metabolic disorders Nature 2006 444 860 867 17167474 10.1038/nature05485

[bib2] Cildir G Akincilar SC Tergaonkar V Chronic adipose tissue inflammation: all immune cells on the stage Trends Mol Med 2013 19 487 500 23746697 10.1016/j.molmed.2013.05.001

[bib3] McNelis JC Olefsky JM Macrophages, Immunity, and Metabolic Disease Immunity 2014 41 36 48 25035952 10.1016/j.immuni.2014.05.010

[bib4] Tilg H Diehl AM Cytokines in alcoholic and nonalcoholic steatohepatitis N Engl J Med 2000 343 1467 1476 11078773 10.1056/NEJM200011163432007

[bib5] Galkina E Ley K Immune and inflammatory mechanisms of atherosclerosis (*) Annu Rev Immunol 2009 27 165 197 19302038 10.1146/annurev.immunol.021908.132620 PMC2734407

[bib6] Andersson U Erlandsson-Harris H Yang H Tracey KJ HMGB1 as a DNA-binding cytokine J Leukoc Biol 2002 72 1084 1091 12488489

[bib7] Sims GP Rowe DC Rietdijk ST Herbst R Coyle AJ HMGB1 and RAGE in inflammation and cancer Annu Rev Immunol 2010 28 367 388 20192808 10.1146/annurev.immunol.021908.132603

[bib8] Wang H Li W Goldstein R Tracey KJ Sama AE HMGB1 as a potential therapeutic target Novartis Found Symp 2007 280 73 85discussion 85–91, 160-4. 17380789

[bib9] Kalinina N Agrotis A Antropova Y DiVitto G Kanellakis P Kostolias G Increased expression of the DNA-binding cytokine HMGB1 in human atherosclerotic lesions: role of activated macrophages and cytokines Arterioscler Thromb Vasc Biol 2004 24 2320 2325 15374849 10.1161/01.ATV.0000145573.36113.8a

[bib10] Kanellakis P Agrotis A Kyaw TS Koulis C Ahrens I Mori S High-mobility group box protein 1 neutralization reduces development of diet-induced atherosclerosis in apolipoprotein e-deficient mice Arterioscler Thromb Vasc Biol 2011 31 313 319 21088249 10.1161/ATVBAHA.110.218669

[bib11] Morton GJ Kaiyala KJ Fisher JD Ogimoto K Schwartz MW Wisse BE Identification of a physiological role for leptin in the regulation of ambulatory activity and wheel running in mice Am J Physiol Endocrinol Metab 2011 300 E392 E401 21062956 10.1152/ajpendo.00546.2010 PMC3043625

[bib12] Schreyer SA Vick C Lystig TC Mystkowski P LeBoeuf RC LDL receptor but not apolipoprotein E deficiency increases diet-induced obesity and diabetes in mice Am J Physiol Endocrinol Metab 2002 282 E207 E214 11739102 10.1152/ajpendo.2002.282.1.E207

[bib13] Lewis KE Kirk EA McDonald TO Wang S Wight TN O'Brien KD Increase in serum amyloid a evoked by dietary cholesterol is associated with increased atherosclerosis in mice Circulation 2004 110 540 545 15277327 10.1161/01.CIR.0000136819.93989.E1

[bib14] Montes VN Turner MS Subramanian S Ding Y Hayden-Ledbetter M Slater S T cell activation inhibitors reduce CD8+ T cell and pro-inflammatory macrophage accumulation in adipose tissue of obese mice PLoS ONE 2013 8 e67709 23844072 10.1371/journal.pone.0067709 PMC3699637

[bib15] Subramanian S Han CY Chiba T McMillen TS Wang SA Haw A 3rd Dietary cholesterol worsens adipose tissue macrophage accumulation and atherosclerosis in obese LDL receptor-deficient mice Arterioscler Thromb Vasc Biol 2008 28 685 691 18239153 10.1161/ATVBAHA.107.157685 PMC2767166

[bib16] O'Brien KD McDonald TO Kunjathoor V Eng K Knopp EA Lewis K Serum amyloid A and lipoprotein retention in murine models of atherosclerosis Arterioscler Thromb Vasc Biol 2005 25 785 790 15692094 10.1161/01.ATV.0000158383.65277.2b

[bib17] den Hartigh LJ Wang S Goodspeed L Ding Y Averill M Subramanian S Deletion of serum amyloid a3 improves high fat high sucrose diet-induced adipose tissue inflammation and hyperlipidemia in female mice PLoS ONE 2014 9 e108564 25251243 10.1371/journal.pone.0108564 PMC4177399

[bib18] Romeo GR Lee J Shoelson SE Metabolic syndrome, insulin resistance, and roles of inflammation—mechanisms and therapeutic targets Arterioscler Thromb Vasc Biol 2012 32 1771 1776 22815343 10.1161/ATVBAHA.111.241869 PMC4784686

